# Sources of Contraceptive Commodities for Users in Nigeria

**DOI:** 10.1371/journal.pmed.0020306

**Published:** 2005-10-18

**Authors:** Boniface A Oye-Adeniran, Isaac F Adewole, Augustine V Umoh, Adesina Oladokun, Abidoye Gbadegesin, Kofo A Odeyemi, Ekanem E Ekanem

**Affiliations:** **1**Department of Obstetrics and Gynaecology, College of Medicine, University of Lagos (CMUL), Lagos, Nigeria,; **2**Department of Obstetrics and Gynaecology, College of Medicine, University of Ibadan, Ibadan, Nigeria,; **3**Department of Obstetrics and Gynaecology, University of Uyo Teaching Hospital (UUTH), Uyo, Akwa Ibom State, Nigeria,; **4**Department of Obstetrics and Gynaecology, Ayinke House, Lagos State University Teaching Hospital (LASUTH), Ikeja, Nigeria,; **5**Department of Community Health, College of Medicine, University of Lagos (CMUL), Lagos, Nigeria; Royal Free and University College LondonUnited Kingdom

## Abstract

**Background:**

Understanding the sources of contraceptive commodities is an important aspect of the delivery of family planning services and is required by planning programme managers for strategic planning purposes. Findings from the 2003 Nigeria Demographic and Health Survey have previously showed that the private sector was the most frequently reported source of contraceptive supply, providing contraception to two and a half times as many women as the public sector. We conducted a community-based study to examine further the sources of contraceptive commodity for users in Nigeria with a view to identifying their preferences for distribution centres. This information would be useful to improve commodity distribution and to build the necessary capacity for satisfactory delivery of contraceptives.

**Methods and Findings:**

A multi-stage random sampling technique was used. A state was randomly selected to represent each of the four health zones in Nigeria. Two local government areas (LGAs) were then selected representing both urban and rural areas. Ten enumeration areas were subsequently selected from each LGA. Of the 2,001 respondents aged 15–49 years, 1,647 (82.3%) were sexually active, out of which 244 were found to be using contraceptive methods at the time of the study, giving a contraceptive prevalence of 14.8%. The commonest source of information on contraceptives was through friends (34%), followed by the radio (11.5%) and husbands (10.2%). Most respondents procured their contraceptives from chemist/patent medicine shops (19.7%), while only 0.8% obtained them from designated family planning clinics. The younger groups in this study (15–24 years), single people, Catholics, and Muslims, showed a greater preference for chemist/patent medicine shops for their sources of contraceptives. The older groups and married respondents, however, made use of government and private hospitals to obtain their contraceptives.

**Conclusion:**

Strategies to increase contraceptive use must take into consideration these identified sources of contraceptives with a view to enhancing the quality, quantity, and variety of methods available, and to building capacity for effective service delivery. There is also a need to encourage the establishment of adolescent-friendly clinics where young people can go for counselling and obtain contraceptives of their choice, including emergency contraceptive pills.

## Abstract 2

In Nigeria contraceptives are available from a wide range of sources, but few, especially young, people get the contraceptives or advice on contraception from official clinics.

## Introduction

Contraceptive methods are available in different forms—oral contraceptive pills, injectables, foaming tablets, intrauterine devices (IUDs), cream, jelly, implants, and barriers in the form of condoms for females and males. While some of these are available only in family planning clinics and hospitals, and many must be given only by trained providers (such as IUDs and implants), others are available in diverse places.

The settings of conventional family planning clinics may discourage certain groups of people from utilizing the facilities, and may be one reason for reported high awareness but low usage of contraceptives [1–3]. Knowledge of clients' source preferences can help to ensure that the commodities are provided where they are most likely to be accepted. Such knowledge has been very helpful in social marketing for such commodities as condoms, and to some extent, oral contraceptives [4–6]. In some countries, such as Zimbabwe, community-based distributors bring the commodities closer to users [4,5]; in other situations, volunteers may be employed. Getting commodities, especially barrier methods such as condoms, closer to the people in acceptable, culturally sensitive, and friendly ways that encourage their use could not be more urgent than it is now, with the need to contain the HIV/AIDS pandemic.

The sources of contraceptive commodities cannot only limit the number of available methods; they can also affect the kind of information clients receive on the methods chosen. Incorrect information leads to unsatisfactory usage, which can ultimately result in high discontinuation and failure rates.

Understanding the sources of contraceptive supplies and the preferences of users will help in strengthening existing networks and planning strategies to address areas of deficiency in a bid to encourage their use. We aimed to examine the sources of contraceptive commodities for users in Nigeria with a view to identifying their preferred sources and planning appropriate interventions where necessary.

## Methods

### Sample Size

This was primarily a descriptive study. The aim was to estimate any given population parameter with a specified level of precision and confidence. The level of confidence was specified as 95% and the tolerable error margin was 5%. Several specifications for *p* were made based on the study objectives. The largest sample size that satisfied all objectives was used. The expression for cross-sectional descriptive studies was *n = Z* ^2^(*100* − *p*)*p*/*δ* ^2^, and a sample of 323 respondents was needed. The sample sizes were adjusted further to compensate for an expected nonresponse rate of 20%, thus the final minimum sample size was 480 per state.

### Sample Locations

Nigeria is divided into states, each of which is divided into local government areas (LGAs). Each LGA is further divided into enumeration areas (EAs). Four states—Anambra from the southeast, Oyo from the southwest, Kaduna from the northwest, and Bauchi from the northeast—were randomly selected for the study, one each from four health zones. In each state, two LGAs (one urban and one rural) were randomly selected. EAs, geographic clusters that have been clearly demarcated by the National Population Commission, served as the primary sampling unit in the LGAs. A systematic sampling approach was used to select ten EAs from each LGA.

### Interviewing

Interviewers were females who had had previous experience in community-based health-related research. A central training was organized for the interviewers that included interviewing techniques, detailed explanations of each of the questions, eligibility and selection of respondents, and role-plays. The questionnaire was then pretested for comprehensibility, appropriateness of language, sensitivity of questions, and average duration for administration. Ethical approval was obtained from the Ethical Committee of the Lagos University Teaching Hospital, Lagos, Nigeria. The interviewers obtained the consent of each respondent.

A multi-stage cluster sampling design was employed to select the respondents. From each LGA, 250 women in the reproductive age group (15–49 years) were included in the survey. In each of the ten EAs (for which a sample size of 25 women was needed), a random starting point was determined in the field by the supervisor using a community landmark such as village square, church, or a mosque. Eligible respondents were consecutively recruited and interviewed until the required sample size for the selected EA was achieved. Only one eligible respondent was interviewed per household. If a household had more than one eligible respondent, only one was randomly selected. A total of 2,001 respondents were interviewed, and these were fairly evenly distributed between rural and urban areas.

### Data Analysis

The EPI Info (6.04d) [7] was used for data entry, validation, cleaning, and analysis. The CHECK option was used to minimize errors arising from inconsistent and illegal entries. Subsequently, the data were re-entered by an independent data entry clerk, and the VALIDATE menu was used to validate these entries. Frequency distribution tables and graphs were generated for all categorical variables. Means and standard deviations and other descriptive measures were determined for quantitative variables such as age of respondent, age at first use of contraception, etc. The chi-square statistic was used for the comparison of proportions and for evaluating association in contingency tables.

## Results

Of 2,001 respondents aged 15–49 years, 1,647 were sexually active and 244 were using contraceptive methods at the time of the study, giving a contraceptive rate of 14.8% for all methods in this age group. Of these, 166 were using modern methods (e.g., oral contraceptives, IUDs, injections, implants, foam, and jelly), giving a usage rate of modern methods of 10.1%.

The commonest sources of information on contraceptives were friends (34.0%), followed by the radio (11.5%). Only about 4.1% reported to have received their information through television. While mothers contributed about 4.1% to source of contraceptive information, fathers did not seem to play any part, as shown by the zero value in Table 1.

**Table 1:**
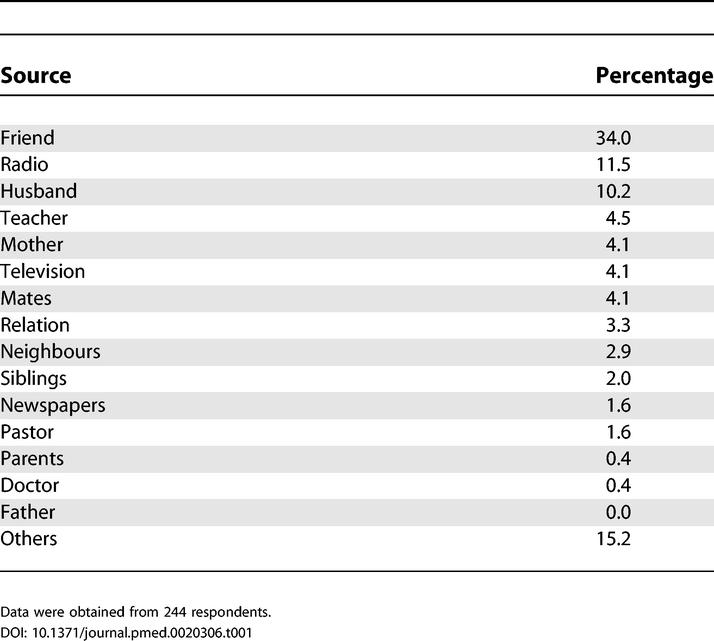
Sources of Contraceptive Information among Sexually Active Respondents

The oral contraceptive pill was the best-known contraceptive method among sexually active respondents (65%), although injectable forms (60.2%), the condom (53.7%), and the IUD (53.3%) were also relatively well known. Most of the respondents were aware of more than one method of contraception (Table 2).

**Table 2:**
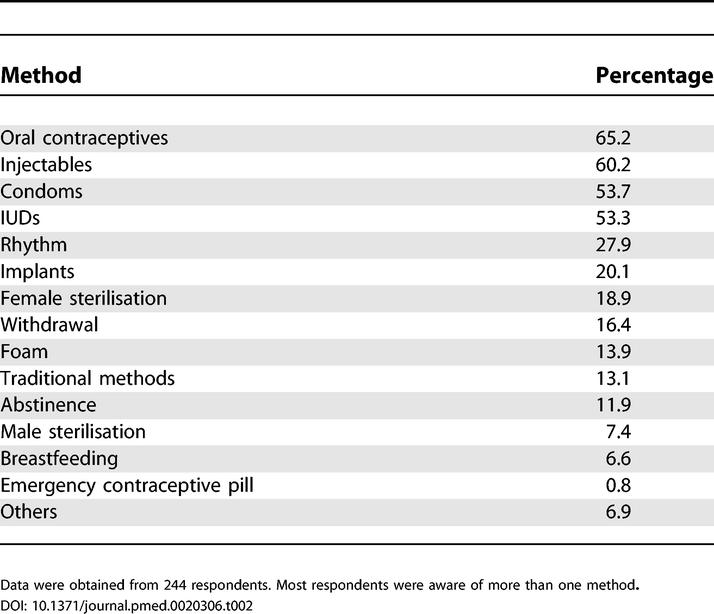
Awareness of Contraceptive Method among Sexually Active Respondents

Most of the respondents who used contraception procured it from chemist/patent medicine shops (16.0%), while the designated family planning centres were rarely patronised (0.8%) (Table 3).

**Table 3:**
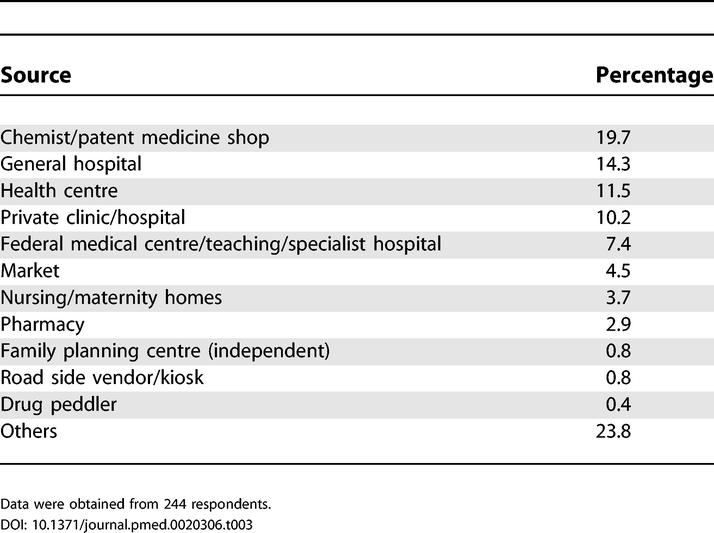
Sources of Contraceptive Commodities

Further evaluation showed that oral contraceptives and condoms were procured from chemist shops, while IUDs were procured mainly from hospitals. Family planning clinics were the only source of the injectables, and even then, in only 0.8% of cases was this method used. Others, including husband, home, church, friends, and herbalist were the main sources of the traditional methods (Table 4).

**Table 4:**
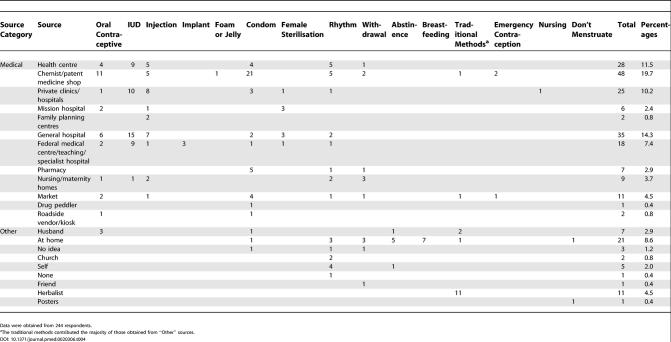
Sources of Contraceptive Commodities by Method

At the place of procurement, the client and her partner largely made the choice of the specific method. Service providers rarely made the choice of method.

Most married respondents preferred to procure their commodities from government hospitals (46.6%), whereas most single respondents (49.1%) procured their commodities from chemist/patent medicine shops (Table 5).

**Table 5:**
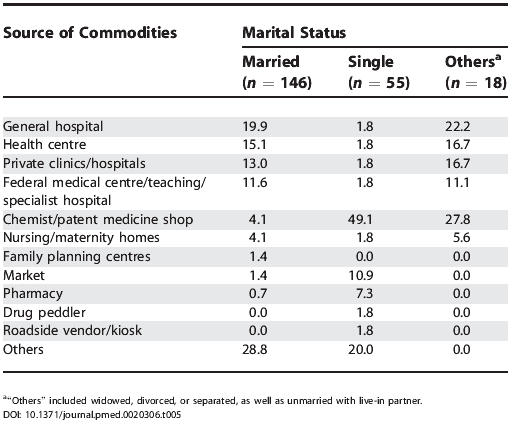
Marital Status and Source of Commodities by Percentage

Most Catholic adherents (59.7%), avoided public health institutions (defined here as health centers and general and teaching hospitals), and no Catholics went to a family planning clinic to obtain any method (Table 6). Muslims, in contrast, made use of health centres and family planning clinics, whereas they did not report obtaining contraceptives from drug peddlers or roadside vendors at all.

**Table 6:**
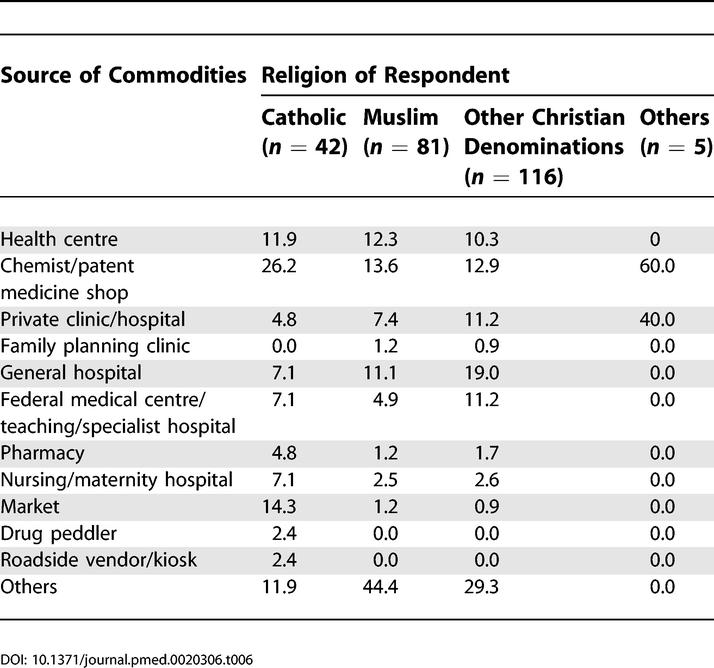
Religion of Respondent and Source of Commodities by Percentage

Young persons (15–24 years) showed a greater preference for chemist/patent medicine shops to obtain their contraceptives. The older groups, however, made use of general or private hospitals to obtain their contraceptives (Table 7).

**Table 7:**
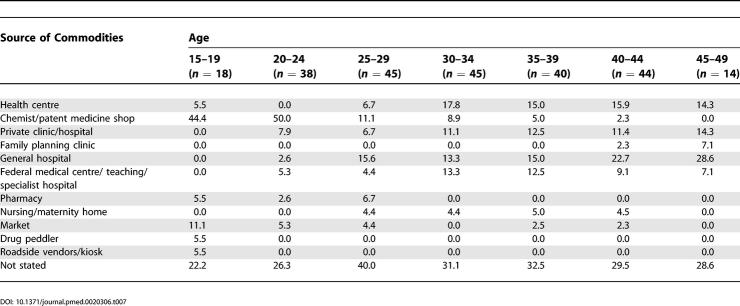
Age of Respondents and Source of Commodities by Percentage

## Discussion

The value of the effective use of contraceptive methods in preventing unwanted pregnancy in our community has been highlighted in a previous study [8]. The high level of contraceptive awareness and low level of usage is also established [2,9]. Various reasons have been advanced for these findings, ranging from cultural barriers, religion, cost, husband/partner's refusal, availability, accessibility, and fear of side effects.

Our study showed that majority of the respondents (34%) got their information through friends, 11.5% heard it over the radio, and 10.2% heard it from their husbands. This result corroborates the findings in the previous study [8]. However, information obtained through friends might be either incomplete or incorrect. Very few of the respondents (0.4%) got their information from the doctor; none got it from their fathers. Neighbours, relations, mates, and siblings were other sources of contraceptive information to the respondents. These results point to a lack of reliable and correct contraception information in these communities.

Of the various sources of contraceptive commodities, chemist/patent medicine shops (16.0%), general hospitals (13.9%), and health centres (11.1%) were the most frequently mentioned. This pattern is also reported amongst sexually active young urban Nigerians [10], in contrast to findings in India, where 75% of users of modern contraceptive methods obtain them from government sources [11].

In this study, the majority of respondents obtained their oral contraceptives and condoms from chemists in the private sector. This pattern is similar to that found in the 2003 Nigeria Demographic and Health Survey [12]. However, this survey appears to have included both the chemist and pharmacy together, as there was no separate category for chemist. Studies in Ghana and Kenya have also shown that these commodities are obtained mainly from the private sector [13,14]. In Zimbabwe and Tanzania, however, the majority of users obtained oral contraceptives and condoms from the public sector [15,16]. This pattern has been the result of strong government involvement in the provision of family planning services [16]. Ramesh and colleagues found that in India, 75% of users of modern contraceptive methods obtained them from government sources [11]. In contrast, in our study, overall the single most frequently used source of contraceptives was the chemist/patent medicine shop (19.7%).

In this study, very few respondents (0.8%) obtained their contraceptive commodities from family planning clinics, echoing a similar trend reported in Ghana, where there is also a general shift from public to private sources for contraceptive commodities [13]. This is a worrisome trend in the role of the public health facility established with the purpose of providing contraceptives in addition to general health care to the public. Improving general staff attitude toward young people, as well as the breadth and accuracy of information given to seekers of contraceptives, are factors that must be examined. In Nigeria, the Campaign Against Unwanted Pregnancy is promoting youth-friendly reproductive health services in a bid to encourage contraceptive use among young persons and to improve their well-being. As others patronise hospitals for such sources as IUDs, injectables, and sterilisations, strengthening departments and improving staff training in hospitals that provide these services may go a long way in improving their utilisation for contraceptive choices. Current family planning centres may then have to be incorporated into hospital departments.

A striking finding in this study was the influence of marital status on source of contraceptives. We discovered that married respondents obtained their contraceptive commodities most frequently from general hospitals (19.9%), health centres (15.1%), private clinics or hospitals (13%), and federal medical centres/teaching/specialist hospitals (11.6%), but only 4.8% of them utilised chemist/patent medicine and pharmacy shops. In contrast, single respondents most often (49.1%) relied on chemist/patent medicine shops. It is probable that married persons requiring contraception are more likely to require longer-lasting methods such as those that may be provided in hospitals, e.g., IUDs and injectables. Young unmarried persons, in contrast, are more likely to use oral contraceptives and condoms, which are obtainable over the counter and from the chemists.

The distinction between chemist/patent medicine and pharmacy shops is important in terms of information about contraception, because whereas a qualified pharmacist manages the pharmacy shop, a trader who has little or no knowledge about the commodities usually manages the chemist/patent medicine shop. In our community, single women are not culturally accepted at conventional family planning centres. So, married respondents obtain their contraceptive commodities from recognised, properly staffed family planning clinics in hospitals, and therefore are likely to benefit from good counselling. Single women, however, because they obtain their contraceptives mostly from chemist/patent medicine stores, where the provider is a non-professional, may not be as likely to receive adequate counselling.

Our data show that single persons are more likely to use oral contraceptives and condoms, which are obtainable over the counter and from the chemist. Ensuring adequate supplies of condoms is especially important, because they also have a role of prevention of sexually transmitted diseases, including HIV/AIDS. Of note is the proportion of single women (10.9%) who used market places as their sources of commodities. The same group would patronise drug peddlers (1.8%; Table 5). This observation again is very relevant in planning intervention strategies for promoting the use of contraceptives. Efforts directed at mass awareness campaigns and establishment of client-friendly facilities should correct this problem.

Religion and denomination have an important influence on contraceptive usage. While the Catholics got their contraceptives mostly from chemist/patent medicine shops (26.2%), market (14.3%), and health centres (11.9%), most other Christians got their methods from general hospitals (19.0%), teaching hospitals (11.2%), private clinics/hospitals (11.2%), and only 12.9% from chemist/patent medicine shops. Catholics' patronage of chemist/patent medicine shops and market places for contraceptives may be connected to their rejection of the use of modern contraceptive methods except the rhythm method.

Muslims obtained contraceptives from chemist/patent medicine shops (13.6%), health centres (12.3%), and general hospitals (11.1%). They most often (44%) reported obtaining contraceptives from “other” sources. Muslims may use chemist/patent medicine shops as the single most common source of contraceptive commodity because of the reported high disapproval rate of the religion to contraceptive use [8].

The age of the respondent was also very important in the source of contraceptive commodity. Most adolescents (44.4%) used chemist/patent medicine shops. However, from age 25 years, there is a greater attempt to obtain contraceptives from general/private hospitals. This observation might be largely due to the societal and cultural disapproval of sex before marriage, the group to which most of these young persons belong. The popular contraceptive commodities in the younger age groups (condom and pills) were also available over the counter. This information is particularly relevant since this group of young persons (15–24 years) is the one with greater incidence of unwanted pregnancy and unsafe abortion [3].

In conclusion, this study has demonstrated that information and counselling on the various types of contraceptive methods in Nigeria are likely to be inadequate, as shown by the sources of information for most respondents and the places of procurement of the commodities. Strategies to increase contraceptive use will have to take into consideration these identified sources of contraceptive commodities, with a view to increasing the number of methods available, enhancing the variety of sources where different contraceptives can be obtained, and improving delivery of correct and adequate information about choices and usage. There is a strong need to carry out a critical appraisal of the role of the proprietors and operators of those shops that the most vulnerable groups patronise. Operators may require training on counselling techniques to make them not only relevant but also effective in provision of contraceptive services to the community. There is also a need to encourage the establishment of youth-friendly centres where young persons can go for counselling as well as to obtain contraceptive methods, including emergency contraceptive pills. Steps taken in these directions should promote increase contraceptive use.

**Patient Summary**

**Background**

Easy access to different methods of contraception and to accurate information about how to use them correctly is essential if people are to benefit from them. There are many places that contraception and advice can be obtained from, ranging from hospital family planning clinics to informal information from friends and families.

**Why Was This Study Done?**

There is relatively little information on where users in the developing world get their advice about contraception, or where they actually obtain the contraceptives. It is important to have such information, as it allows planning for provision of services, particularly for groups such as adolescents, who rarely seek formal health information.

**What Did the Researchers Do and Find?**

They interviewed 2,001 people from four different states of Nigeria using a questionnaire. They found that 1,647 of these people were sexually active, but only 244 were using contraception. A third of people got their information on contraceptives from friends, and another 10% from the radio. Very few people—less than 1%—got their contraceptives from hospital or clinics, and these were mostly older married people; around 20% of people got them from chemist/patent medicine shops.

**What Do These Findings Mean?**

Overall, there is a worryingly low level of contraceptive use. Most people get information on contraceptives from informal sources, and such information may not always be appropriate for them. Most people also prefer to get the contraceptives themselves from non-official sources.

**Where Can I Get More Information Online?**

The Association of Reproductive Health Professionals has many pages of information for patients:

http://www.arhp.org/patienteducation/index.cfm

The US Centers for Disease Control and Prevention has a page of information on contraception globally:

http://www.cdc.gov/reproductivehealth/Global/index.htm

## Abbreviations

EA: enumeration area
IUD: intrauterine device
LGA: local government area
